# Meteorological Register

**Published:** 1835-04-01

**Authors:** 


### METEOROLOGICAL REGISTER,

FROM DECEMBER 1, 1834, TO FEBRUARY 28, 1835.

By Messrs. HARRIS and Co., Mathematical Instrument Makers, 50, High Holborn.

| | Thermometer. | | Barometer. | | De Luc's Hygrometer. | | Winds. | | Atmospheric Variations. | | |
|---|---|---|---|---|---|---|---|---|---|---|---|
| | *max.* | *min.* | 9 *a.m.* | 10 *p.m.* | 9*a.m.* | 10*pm.* | 9 *a.m.* | 10 *p.m.* | 9 *a. m.* | 2 *p.m.* | 10 *p.m.* |
| Dec. 1 to 7 | 52 | 41 | 30.22 | 29.08 | 81 | 76 | SSW | WSW | fine | fine | showery |
| 7 to 14 | 51 | 30 | .37 | 30.11 | 81 | 76 | WSW | NE | fine | fine | foggy |
| 14 to 21 | 47 | 37 | .36 | 29.16 | 84 | 79 | NNE | NNW | fine | rain | cloudy |
| 21 to 31 | 55 | 36 | .31 | .60 | 88 | 78 | NNW | ENE | fine | foggy | rain |
| Jan. 1 to 7 | 47 | 21 | .45 | .97 | 85 | 74 | NNE | ENE | cloudy | fine | foggy |
| 7 to 14 | 51 | 28 | 29.90 | .42 | 88 | 80 | SSW | SW | fine | fine | rain |
| 14 to 21 | 49 | 26 | 30.03 | .10 | 91 | 80 | SW | NW | rain | fine | snow |
| 21 to 31 | 51 | 36 | .17 | .85 | 87 | 74 | WSW | SSW | cloudy | fine | foggy |
| Feb. 1 to 7 | 52 | 36 | .20 | .59 | 76 | 55 | WSW | SW | cloudy | fine | fine |
| 7 to 14 | 51 | 30 | .18 | .53 | 62 | 52 | WSW | SW | cloudy | mist | fine |
| 14 to 21 | 52 | 31 | .99 | .01 | 62 | 54 | WSW | SW | cloudy | fine | rain |
| 21 to 28 | 50 | 31 | .97 | .01 | 57 | 52 | SW | S | cloudy | rain | rain |

The quantity of Rain fallen in December, 88 100ths of an inch.

The Rain for January cannot be stated, owing to frost. In February, the Rain-gauge was blown down, and broken: consequently, the Rain cannot be given for that month.

